# In-Field and Early Detection of *Xylella fastidiosa* Infections in Olive Using a Portable Instrument

**DOI:** 10.3389/fpls.2018.02007

**Published:** 2019-01-18

**Authors:** Federico Martinelli, Annalisa Marchese, Antonio Giovino, Francesco Paolo Marra, Isabella Della Noce, Tiziano Caruso, Abhaya M. Dandekar

**Affiliations:** ^1^Department of Agricultural Food Forest Sciences, University of Palermo, Palermo, Italy; ^2^Council for Agricultural Research and Economics (CREA), Research Centre for Plant Protection and Certification (CREA-DC), Bagheria, Italy; ^3^Hyris Limited, Milan, Italy; ^4^Department of Plant Sciences, University of California, Davis, Davis, CA, United States

**Keywords:** in-field detection, olive quick decline syndrome, olive, portable instrument, *Xylella fastidiosa*

## Olive Quick Decline Syndrome (OQDS)

*Xylella fastidiosa* subsp. *pauca* (Xfp) is a gram-negative pathogenic bacteria responsible for serious diseases (Purcell, 2013) that inflicts considerable economic loss (Li et al., 2007; Luvisi et al., 2017). The pathogen has been linked to olive quick decline syndrome (OQDS). This devastating olive disease was first observed in Salento (Apulia, southeastern Italy) in 2009. Infected trees respond to Xfp infection with scattered desiccation of twigs and small branches in the upper crown, which extend to the rest of the canopy, showing the characteristic blight effect. The disease causes tree death within a few years from the onset of symptoms (Martelli, 2016). The primary agronomic procedure for counteracting the infection is by heavy pruning to stimulate new growth (Martelli et al., 2016). However, this does not prevent the withering and desiccation of upper vegetation in the infected tree. Lignin deposition increases the tolerance of some hosts to *Xylella fastidiosa*. Elevated concentration of quinic acid, a lignin precursor, less concentration of hydroxytyrosolglucoside and the up-regulation of cinnamoyl-CoA reductase and polyphenol oxidase were observed in the most tolerant olive cultivar, Leccino (Sabella et al., 2018).

In this opinion article, we explore the use of a portable instrument to detect OQDS, based on the host responses at the transcript level. This approach was proposed previously to detect Huanglongbing, a severe disease affecting Citrus worldwide (Dandekar et al., 2010; Martinelli et al., 2014b). These innovative methods of plant disease detection had been reviewed recently (Martinelli et al., 2014a).

## A Portable Instrument To Detect Early Olive Quick Decline Syndrome

**Figure 1:**
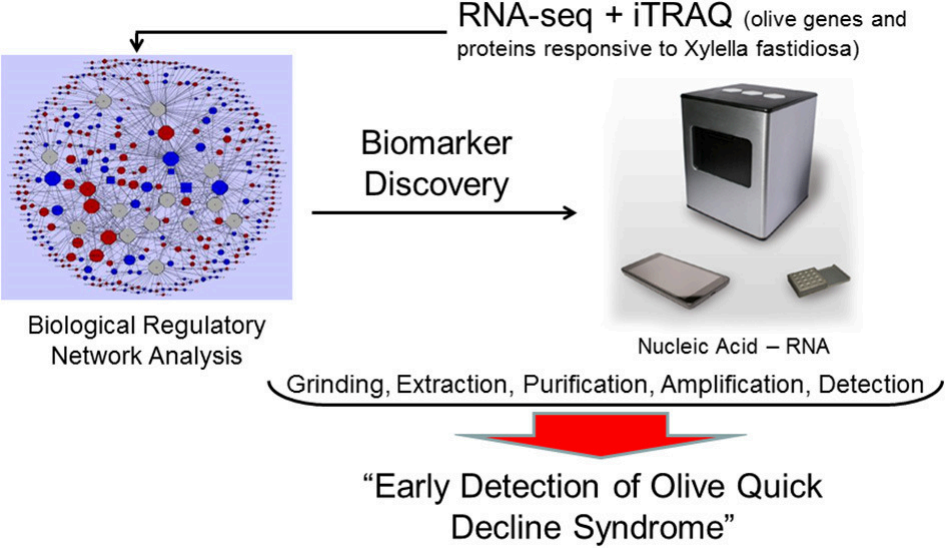
Multi-omic approach to detect early olive quick decline syndrome with portable instruments in the field. RNA-seq and proteomic iTRAQ approaches can identify host and pathogen biomarkers that can be analyzed directly in the field by a portable instrument for rapid in-field diagnosis of *Xylella fastidiosa* infections.

Molecular tools for early in-field detection of *Xylella fastidiosa* infections have been developed (Baldi and La Porta, 2017; Chiriacò et al., 2018). A new portable instrument, the bCUBE, marketed by Hyris, Ltd., can effectively detect plant diseases. We believe that this instrument could also be used to detect olive responses to *Xylella fastidiosa* infections. The portable instrument analyzes mRNA using in-field qRT-PCR (Figure 1). Analysis of expression could be conducted on leaf, fruit, or bark tissues, targeting genes linked with OQDS syndrome. RNA can be extracted in the field with a quick procedure developed to detect other plant pathogens with bCUBE. Results are provided in ~ 45 min. The already-developed software and hardware can be used to optimize the instrument to detect expression of host Xfp-regulated genes. A bioinformatics analysis can be conducted through meta-analysis of published gene expression data related to Xf-host interactions. There is already a wide analysis of transcriptomic responses to this pathogen in different crops. Raw data deposited in public databases can be re-analyzed using the same pipeline to determine which differentially regulated genes are most commonly expressed. This analysis would show how much currently available information can be used to develop a portable instrument that detects these Xfp-regulated biomarkers. Implementation and validation tests would analyze the same tissues simultaneously in the field (with the portable instrument) and in the lab (traditional qRT-PCR) to verify the consistency of the field results provided by the instrument and to validate and optimize its use. We believe that this instrument should be tested in an olive grove where OQDS syndrome is already present by analyzing trees at different disease stages: healthy, apparently healthy, and both asymptomatic and symptomatic tissues of symptomatic trees.

## A Transdisciplinary Approach to Identify Olive Xfp-Responsive Genes as Biomarkers for In-Field Detection

A broad range of environmental stress biomarkers (transcripts, miRNAs, proteins, or metabolites) have been identified through integrated omic approaches (Natali et al., 2007; Martinelli and Tonutti, 2012; Tosetti et al., 2012). As the pathogen is not evenly distributed in an infected plant's tissues, identifying specific genes induced by the pathogen in a range of tissues is necessary to discover tissue-specific host biomarkers (Giovino et al., 2015). The pathogen's presence is perceived by the infected plant before the pathogen DNA concentration exceeds the detection limit of traditional real-time systems. Such analyses will clarify pathogenetic disease mechanisms by clarifying the relationships between cause and effect. It is still unclear how the pathogen contributes to disease manifestation and how other environmental factors contribute. The proposed approach would also provide molecular targets to develop resistant genotypes as it refines existing technology into a novel portable instrument for real-time disease detection in orchards. It links genomics to technology that enhances the discovery of disease-specific biomarkers and closes the gaps in conventional disease scouting and molecular diagnostic methods. Disease-specific biomarkers will be discovered through genomic and network analysis of host and pathogen responses. Close integration of different activities will combine technology and research. Graft-infected olive plants should be inoculated. Five to ten plants should be sampled in each category and tissues should be collected from the top branches at 7, 21, 35, and 60 days after infection. When symptoms appear, infection status should be verified by PCR. In addition to the longitudinal experiment, a transcriptomic analysis of naturally infected trees at different disease stages should be performed. The first category of trees (healthy) might be available in a nearby olive grove where the pathogen has not yet been detected. If it is subjected to similar climatic, agronomic and pedological conditions to infected trees, these trees can represent control uninfected conditions. The second category of trees is present in the infected field, but is apparently healthy and without detectable pathogen. Since we cannot exclude the presence of the pathogen, these trees provide a different control because later diagnostic analysis might detect infection. The third category of trees is clearly symptomatic, PCR-positive or ELISA-positive. The discovery of potential markers linked with early olive response to Xfd infection may be performed using RNA-seq approaches and Illumina technology (Hiseq 2000). Some transcriptomic studies at different symptomatic stages have been conducted in *Vitis* and *Citrus* crops infected by *Xylella fastidiosa* (Rodrigues et al., 2013; Zaini et al., 2018). Some genes are commonly expressed in *Vitis* and *Citrus* responses to *Xylella fastidiosa* (Table 1). Interestingly, some of these genes are differentially regulated by *Xylella fastidiosa* in susceptible and tolerant genotypes, highlighting their possible use as biomarkers for tolerance. These data provide a good starting point to test the instrument's ability to identify olive orthologs of these Xf-regulated genes. Our meta-analysis identified some good candidate genes: a leucine-rich repeat protein kinase family protein, *MYB66*, chitinase A, a trypsin inhibitor family protein, expansin A4 and some auxin-responsive and gibberellin-regulated genes (Table 1). Xf-regulated genes commonly present in the two transcriptomic works conducted in *Vitis* and olive might be also of interest (Giampetruzzi et al., 2016; Zaini et al., 2018; Table 2). Some key cell wall modification genes that are potentially involved in pathogen signaling responses were observed: a pectin-lyase, a laccase and a poligalacturonase. This latter gene was commonly regulated in transcriptomic data obtained from all three species (*Citrus, Vitis*, and olive). This preliminary meta-analysis does not replace a longitudinal RNA-seq analysis focusing on early asymptomatic stages of infection, required to render the instrument highly sensitive and reliable. RNA-seq analysis cannot be exploited fully unless the quality of the olive genome sequence is improved. At the moment, only a draft version of the Farga genotype is available (Cruz et al., 2016). However, a more advanced version of the olive genome is expected soon due to ongoing international initiatives by an Italian-Spanish consortium that began in 2009. With the progressive reduction of sequencing costs, an improved olive genome is expected soon, allowing improved RNA-seq approaches. Indeed, we believe that the concept of an in-field device to detect host transcriptomic responses to Xfp will push efforts to obtain a high-quality olive genome sequence.

**Table 1:** Common *Citrus* and *Vitis* differentially-expressed genes in response to *Xylella fastidiosa* infection (Rodrigues et al., 2013; Zaini et al., 2018).

**Gene symbol**	**Gene id *Citrus clementina***	**Gene id *Vitis***	***Arabidopsis* best match**	**Gene description**
*LRR-RLK*	Ciclev10014130m	VIT_12s0028g01950	AT3G47570	Leucine-rich repeat protein kinase family protein
*MYB66*	Ciclev10017556m	VIT_17s0000g08480	AT5G14750	myb domain protein 66; transcription factor
*CHIA*	Ciclev10005491m	VIT_14s0066g00610	AT5G24090	chitinase A
*TRYPSIN*	Ciclev10022001m	VIT_17s0119g00280	AT1G17860	Kunitz family trypsin and protease inhibitor family protein
*HSP17*	Ciclev10009756m	VIT_04s0008g01520	AT5G12020	17.6 kda class II heat shock protein
*PAL*	Ciclev10030821m	VIT_16s0039g01300	AT2G37040	Phenylalanineammonia-lyase
ATFXG1^**^	**Ciclev10030947m**	**VIT_01s0127g00870**	**AT1G70370**	**Polygalacturonase 2**
UDP-glycosyltransferase	Ciclev10025462m	VIT_18s0001g12040	AT3G50740	udp-glucosyltransferase 72E1
*ATEXPA4*	Ciclev10012518m	VIT_06s0004g04860	AT2G39700	Expansin A4
*CML37*	Ciclev10002523m	VIT_18s0122g00180	AT5G42380	Calmodulin like 37
Auxin-responsive family protein	Ciclev10015492m	VIT_12s0057g00420	AT5G35735	Auxin-responsive family protein
Gibberellin-regulated family protein	Ciclev10013200m	VIT_08s0007g05860	AT1G74670	Gibberellin-regulated family protein

** *also in common with Citrus*.

**Table 2:** Common differentially expressed genes in *Vitis* (Zaini et al., 2018) and *Olea* in response to *X. fastidiosa* infection (Giampetruzzi et al., 2016).

**Gene symbol**	**Gene ID *Vitis***	**Sequence ID in olive**	***Arabidopsis* best match**	**Gene description**
PR proteins	VIT_09s0018g01670	og_xylem_263402	AT1G79720	Eukaryotic aspartyl protease family protein
PR proteins	VIT_00s0510g00030	og_xylem_275149	AT5G42180	Peroxidasesuperfamilyprotein
NADP-binding Rossmann fold	VIT_08s0007g07520	og_xylem_274898	AT4G13180	NAD(P)-binding Rossmann-fold superfamily protein
		og_xylem_282761		
		og_xylem_353110		
Calcium signaling	VIT_01s0010g03010	og_xylem_182510	AT1G76650	Calmodulin-like 38
Cell wall remodeling	VIT_13s0067g01970	og_xylem_303721	AT5G05390	Laccase 12
Cell wall remodeling	VIT_01s0127g00870	og_xylem_288909	AT1G70370	Polygalacturonase 2^**^
Cell wall remodeling	VIT_01s0127g00400	og_xylem_299947	AT1G60590	Pectinlyase-like superfamily protein
		og_xylem_304420		
Cell wall remodeling	VIT_08s0040g02070	og_xylem_275695	AT5G03170	FASCICLIN-likearabinogalactan-protein 11
		og_xylem_283555		

** *also in common with Citrus*.

Proteomics may be conducted in parallel with transcriptomics using the iTRAQ method. This approach has been used in *Vitis* (Chakraborty et al., 2016). The use of proteomics is important to validate transcriptomic markers considering that genes may be regulated by post-transcriptional mechanisms. Proteins can be extracted from powdered olive leaf and bark tissues using a highly efficient phenol extraction procedure (Schuster and Davies, 1983). This protocol can extract protein from tissues that contain highly reactive compounds. The extracted proteins can be precipitated using the ProteoExtractTM Protein Precipitation kit (Calbiochem), dehydrated overnight, then re-suspended and subjected to tryptic digestion. The digested peptides can be analyzed using a QExactive mass spectrometer (Thermo Fisher Scientific) coupled with an Easy-LC (Thermo Fisher Scientific) and a nanospray ionization source. The peptides are loaded onto a trap (100 micron, C18 100Å 5U) and desalted online before separation using a reverse-phased column (75 micron, C18 200Å 3U). Data are acquired using data-dependent ms/ms, which has a full scan range of 300–1600 Da and a resolution of 70,000. Raw data are analyzed using X!Tandem and visualized using Scaffold Proteome Software (Version 3.01). Samples are compared to Uniprot databases appended with the cRAP database, which contains common laboratory contaminants. Reverse decoy databases are also applied to the database prior to the X!Tandem searches. This procedure should identify the secreted virulence factor of *Xylella fastidiosa* in olive. Some identified pathogen virulence proteins will be selected for *in planta* expression to validate their virulence phenotype. Synthetic virulence genes should be designed to insure greater in-plant expression. Codons might be optimized by replacing codons that are less frequently used in *Citrus* by DNAworks software (http://mcl1.ncifcrf.gov/dnaworks/). Signal peptides could be analyzed by SignalP 4.0 server (http://www.cbs.dtu.dk/services/SignalP/). N-Glycosylation sites may be predicted using NetNglyc1.0. More than 4,500 proteins, including several pathogen targets, have been identified in *Citrus* leaf tissues infected by Huanglongbing disease, comparing tolerant and susceptible genotypes (Martinelli et al., 2016).

## Conclusions

Efficient pre-symptomatic diagnosis of OQDS relies on the ability to (i) obtain informative biomarkers from *Xylella fastidiosa* and host (olive), and then (ii) detect the biomarkers in a rapid, sensitive, and cost-effective manner. Before the onset of disease symptoms, the expression pattern of these biomarkers and their interplay changes at the early stages of infection. A deep transcriptomic analysis using multiple genotypes is highly desirable to define thresholds of biomarker expression and optimize efficiency. Once biomarkers (genes) are determined, the goal is to design a device that integrates sample preparation, nucleic acid enrichment, and final detection. This translational genomic approach will permit the following impacts: (1) develop and deploy inexpensive electronic instruments to identify Xfp infections at early, pre-symptomatic stages, (2) clarify vector-pathogen-plant interaction to monitor the efficacy of field interventions or treatments, (3) enhance disease scouting through new disease detection and diagnosis tools and improved stakeholder outreach, and (4) adapt the detection and diagnostic tools to different specialty crop diseases.

## Author Contributions

FM conceived the opinion and designed and wrote the article. AM, FPM, TC, AG, ID, and AD approved and contributed to the writing of the manuscript.

### Conflict of Interest Statement

The authors declare that the research was conducted in the absence of any commercial or financial relationships that could be construed as a potential conflict of interest.
